# The dark side of team dynamics: how team differential atmosphere undermines team creativity

**DOI:** 10.3389/fpsyg.2026.1658508

**Published:** 2026-03-25

**Authors:** Yuanzhe Liu, Xiaoqian Qu, Hongzhen Lei

**Affiliations:** International Business School, Shaanxi Normal University, Xi'an, China

**Keywords:** inclusive leadership, multicultural teams, team creativity, team differential atmosphere, Team Empathy, team interaction process

## Abstract

**Introduction:**

Team Differential Atmosphere (TDA)-a climate-level perception of rigid “insider” and “outsider” boundaries created by leaders based on relational closeness-has emerged as an important barrier to creativity in multicultural teams. Although inclusive leadership is widely recognized as a catalyst for innovation, the specific micro-mechanisms by which its opposite, TDA, disrupts team dynamics remain unclear. Drawing on Social Information Processing Theory (SIPT), this study positions TDA as a distinct signal of identity exclusion, distinguishing it from Leader-Member Exchange Differentiation (LMXD).

**Methods:**

Data were collected from 79 innovation teams using a multi-source approach, and a moderated mediation model was developed and tested.

**Results:**

The results indicate that: (1) TDA, as a manifestation of non-inclusive leadership, significantly inhibits team creativity; (2) this inhibition occurs via two distinct pathways: team collaboration (reduced collaborative behaviors) and joint decision-making (impaired information integration); and (3) team empathy, as a key collective emotional resource, can buffer the detrimental effects of TDA on these processes.

**Discussion:**

This study contributes to the literature by distinguishing the “identity threat” signal of TDA from the performance-related signal of LMXD and by clarifying how differential structures can adversely affect team interaction processes and creativity at the team level. It further identifies Team Empathy as a bottom-up resilience resource that buffers these negative effects in multicultural teams.

## 1 Introduction

Driven by the wave of globalization, multicultural teams have become a primary driver of organizational innovation, providing enterprises with rich cognitive resources to address complex challenges and sustain a competitive advantage (van Knippenberg, 2017; Homan et al., 2020). While extant literature predominantly emphasizes inclusive leadership as the key to unlocking the creative potential of these diverse teams (Randel et al., 2018), a critical theoretical tension remains unresolved: in reality, leaders frequently deviate from inclusive principles. Despite the well-documented benefits of fostering belongingness and uniqueness, managers often succumb to relational biases and systematically enact non-inclusive, differentiation-based management (Liu et al., 2009). This structural exclusion directly contradicts the supportive and psychologically safe climate that team creativity heavily relies upon, creating a severe paradox in team dynamics (Shen et al., 2019). To address this underexplored tension, this study focuses on a specific exclusionary leadership phenomenon—the Team Differential Atmosphere (TDA)—and deeply investigates how it acts as a structural barrier to creativity in multicultural teams.

To address the conceptual ambiguity surrounding differential leadership, it is imperative to explicitly articulate the theoretical positioning of TDA in relation to mainstream constructs like Leader-Member Exchange Differentiation (LMXD). Both TDA and LMXD describe structural disparities in leader resource allocation and can similarly trigger feelings of unfairness and team conflict (Hooper and Martin, 2008; Liu et al., 2009). However, they possess fundamentally distinct theoretical foundations and scopes. First, regarding the basis of differentiation, LMXD is rooted in Western social exchange theory and functions as a performance-based, meritocratic logic; it relies on members' competence, work contributions, and task congruence with the leader (Graen and Uhl-Bien, 1995). Conversely, TDA is an indigenous phenomenon deeply embedded in the Eastern cultural context, specifically the Chinese differential mode of association (Chaxu Geju). The foundation of TDA stems from non-work-related relational ties—such as shared hometowns, alumni networks, or nepotism—rather than professional merit (Fei et al., 1992). Second, regarding the scope of impact, LMXD primarily captures specific, dyadic transactional behaviors (Graen and Uhl-Bien, 1995). In contrast, TDA represents a pervasive, team-level shared climate, making its exclusionary impact much broader and deeper across the team's social fabric (Liu et al., 2009; Shen et al., 2019). Through the above distinction, this study explicitly defines TDA not merely as a subtype or a culturally embedded variant of LMXD, but as a completely distinct, climate-level construct wherein members believe their leader establishes rigid “insider” and “outsider” boundaries based on relational favoritism.

According to Social Information Processing Theory (SIPT) (Salancik and Pfeffer, 1978), individuals interpret social cues from their work environment to adjust their behaviors. In multicultural teams, differential treatment based on relational closeness (TDA) serves as a salient identity threat cue. While existing research has predominantly linked TDA to individual-level withdrawal or silence (Chen and Liu, 2020; Liao et al., 2024), its broader impact on collective team interactions remains underexplored. Grounded in established taxonomies of team interaction processes (Marks et al., 2001; Kozlowski and Ilgen, 2006), literature on team innovation indicates that high-performing teams depend heavily on two distinct collective mechanisms: an interpersonal process characterized by supportive teamwork and mutual help (Hoegl and Gemuenden, 2001), and a cognitive process involving the elaboration of diverse information (van Knippenberg, 2017). By signaling explicit “insider” and “outsider” boundaries, TDA is likely to disrupt both processes. To capture this disruption substantively, this study investigates two parallel mediators: Team Mutual Collaboration and Team Joint Decision-Making. Team Mutual Collaboration reflects the breakdown of the interpersonal supportive teamwork, as identity threat reduces marginalized members' motivation for reciprocal helping (Tyler and Blader, 2003; Twenge et al., 2007). Concurrently, Team Joint Decision-Making reflects the failure of cognitive information elaboration, as exclusionary cues prompt defensive information withholding to avoid interpersonal risks (Edmondson, 1999; Connelly et al., 2011). By examining both mediators, this study provides a more comprehensive understanding of how TDA impairs the synergistic processes essential for team creativity.

Furthermore, identifying boundary conditions to mitigate TDA is crucial. While prior research on buffering exclusionary leadership has predominantly focused on distal organizational interventions (e.g., institutional policies) (Mackey et al., 2017) or isolated individual resilience (e.g., psychological capital) (Tepper et al., 2017), scholars suggest that addressing team-level relational stressors requires collective, bottom-up resources to effectively repair fractured social ties (Hobfoll et al., 2018; Bourke et al., 2020). Consequently, we introduce Team Empathy as a moderating mechanism. We conceptualize Team Empathy as a collective resource pool reflecting the team's aggregate capacity for emotional resonance and perspective-taking (Akgün et al., 2015). We selected Team Empathy because research demonstrates its efficacy in dissolving “in-group/out-group” boundaries and alleviating the negative psychological impact of social exclusion (Batson and Ahmad, 2009; Zaki, 2014). Thus, we propose that in highly empathetic teams, the abundance of this collective resource enables members to collaboratively reframe exclusionary signals, thereby buffering the disruptive impact of TDA on mutual collaboration and joint decision-making (Lee and Madera, 2021).

**Figure 1:**
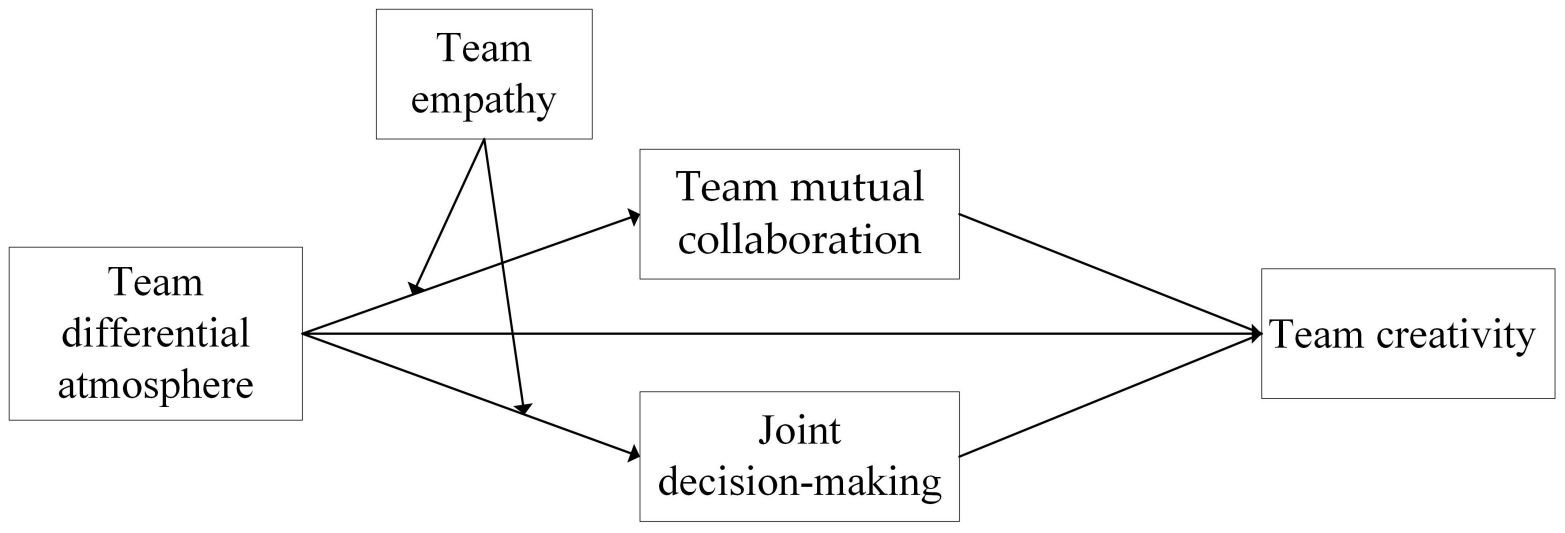
Model framework diagram.

In summary, this study develops a moderated mediation model (see Figure 1) to examine how TDA relates to team creativity through two team-level interaction processes, Team Mutual Collaboration and Team Joint Decision-Making, and how Team Empathy conditions these relationships. Building on Social Information Processing Theory (SIPT), this study advances the literature in three ways. First, it deepens understanding of the culturally specific construct of TDA and places it in dialogue with mainstream LMXD theory; it provides a new theoretical perspective and empirical evidence on the consequences of non-inclusive leadership. Second, it highlights Team Mutual Collaboration and Team Joint Decision-Making as core mediating mechanisms linking TDA to reduced creativity at the team level. Third, it introduces Team Empathy as a key moderating variable, providing a practically relevant lens on how teams can buffer the negative influence of differential atmospheres in multicultural contexts.

## 2 Theoretical and hypotheses

### 2.1 Social Information Processing Theory

Social Information Processing Theory (SIPT) posits that individuals interpret environmental cues to shape their cognitive and affective reactions, which subsequently drive behavioral choices (Salancik and Pfeffer, 1978). In a team context, leader behaviors serve as salient social signals regarding norms and member value. When a leader allocates resources based on non-work-related relational closeness rather than professional merit, this deviation from professional norms forms a salient social information signal. Viewed through the lens of SIPT, TDA functions as an identity threat cue. This threat arises because marginalized members perceive that their core value and status within the team depend heavily on ascribed relational ties rather than their professional contributions, which challenges their professional self-concept.

The processing of such social cues alters the collective interactions required for team creativity. Extensive team literature establishes that synergistic team outcomes depend on two distinct but interrelated foundational processes: an affective, interpersonal process driven by reciprocal support and prosocial motivation (i.e., Team Mutual Collaboration) (Marks et al., 2001), and a cognitive process requiring the deliberate sharing and elaboration of diverse knowledge (i.e., Team Joint Decision-Making) (Hinsz et al., 1997). SIPT explicates how TDA impairs team creativity by negatively affecting both of these pillars. Upon decoding the identity threat cue, members engage in defensive sensemaking. They tend to interpret the social boundaries within the team as impermeable and view the environment as carrying interpersonal risks (Edmondson, 1999; Petriglieri, 2011). To cope with this perceived exclusion, members may experience a depletion of emotional resources and exhibit behavioral withdrawal (Twenge et al., 2007; Shen et al., 2019). This defensive reaction reduces their motivation to engage in mutual collaboration (Tyler and Blader, 2003). Concurrently, concerns about potential interpersonal risks prompt members to adopt defensive cognitive strategies, such as withholding unique information and avoiding open debate (Connelly et al., 2011). This cognitive withholding limits the integration of diverse perspectives necessary for joint decision-making (De Dreu and West, 2001; van Knippenberg, 2017).

Furthermore, SIPT suggests that contextual factors moderate cue interpretation. Within this framework, Team Empathy serves as a critical boundary condition. We conceptualize team empathy as a collective emotional-cognitive resource pool constituted by the additive empathetic capacities of individual members (Akgün et al., 2015). This collective empathetic capacity provides the psychological capital needed to cope with identity threats and intergroup frictions (Batson and Ahmad, 2009). In teams with a high overall level of empathy, the abundant availability of perspective-taking and emotional resonance helps members collectively reframe the leader's exclusionary signals (Zaki, 2014; Lee and Madera, 2021). This collective resource buffers the negative sensemaking process, mitigating the adverse impact of TDA on both the affective willingness to collaborate and the cognitive capacity to make joint decisions (Rego et al., 2007; Bourke et al., 2020).

### 2.2 The distinct nature of TDA *vs*. LMX differentiation

While the introduction has distinguished Team Differential Atmosphere (TDA) from Leader-Member Exchange Differentiation (LMXD) conceptually, this section draws on SIPT (Salancik and Pfeffer, 1978) to further elucidate how these two constructs activate fundamentally divergent psychological processing pathways and behavioral outcomes. Because they signal different sources of disparity, LMXD and TDA trigger distinct sensemaking mechanisms. Building on Social Exchange Theory (Blau, 1964), LMXD is decoded by members primarily as a transactional cue. As it signals a gap in professional exchange, LMXD tends to trigger social comparison processes regarding inputs and outcomes (Festinger, 1954). Extensive literature indicates that even when perceived as unfair, this transactional comparison can sometimes motivate members to adjust their task efforts or compete constructively to improve their standing (Graen and Uhl-Bien, 1995; Vidyarthi et al., 2010; Dulebohn et al., 2012). In contrast, TDA functions as a salient identity and relational cue. By signaling that resource allocation is dictated by ascribed affective closeness rather than professional merit, TDA invalidates the fundamental expectation of “effort-reward” congruence (Adams, 1965). According to Social Identity Theory (Tajfel and Turner, 1986), members interpret these rigid, non-work-related boundaries not as a transactional imbalance, but as an identity threat. Consequently, instead of stimulating competitive motivation, the TDA cue induces social exclusion and psychological withdrawal. This defensive sensemaking systematically impairs members' motivation to engage in mutual collaboration and stifles their cognitive integration in joint decision-making (Liu et al., 2009; Shen et al., 2019).

### 2.3 Team Differential Atmosphere and team creativity

Team Differential Atmosphere (TDA), rooted in the concept of “Differential Mode of Association,” describes a structural disparity where leader resource allocation is perceived as contingent on relational closeness (Liu et al., 2009). We posit that TDA is likely to impede team creativity by creating an adversarial social climate that stifles the cognitive and behavioral processes required for innovation. First, regarding “out-group” members, TDA tends to construct significant informational and emotional barriers (Liu et al., 2009; Shen et al., 2019). When differentiation is based on guanxi rather than merit, excluded members may perceive a lack of procedural justice (Tyler and Blader, 2003). In the multicultural context of this study, such relational segregation can be particularly detrimental, as it risks alienating members who already navigate cultural distinctiveness. Feeling marginalized, these members are less likely to invest their unique cognitive resources into the team, instead adopting defensive behaviors such as withdrawal or silence (Zhao, 2015). This reduction in engagement from a segment of the team directly diminishes the diversity of ideas available for creative synthesis. Second, the negative impact extends to “in-group” members. Although insiders receive preferential access to resources, this privileged status may carry hidden psychological costs that hinder creativity. Drawing on the perspective of status maintenance, insiders often face heightened pressure to preserve their standing in the leader's “circle of trust” (Zhao, 2015; Han et al., 2023). This pressure can induce risk-averse behavior, where insiders stick to safe, conventional solutions rather than proposing novel but risky ideas that might jeopardize their favored status (Shen et al., 2019). Furthermore, the potential friction between insiders and outsiders can create a tense atmosphere that disrupts the overall flow of knowledge (Mayer et al., 2007). Thus, by simultaneously demotivating outsiders and constraining insiders, TDA is expected to suppress the team's overall creative output. In sum, the following hypothesis is proposed:

H1: Team differential atmosphere negatively affects team creativity

### 2.4 The mediating role of Team Mutual Collaboration

Existing research on team dynamics establishes that team functioning relies on two distinct pillars: cognitive information processing and interpersonal processes (Marks et al., 2001; Kozlowski and Ilgen, 2006). Aligning with this framework, Team Mutual Collaboration functions as a critical interpersonal mechanism, characterized by reciprocal helping and prosocial support (LePine et al., 2008). Under the SIPT framework, TDA serves as an identity threat cue that can negatively impact this relational fabric. When members perceive that the leader enforces rigid “insider” and “outsider” boundaries based on personal ties, they are likely to experience a heightened sense of social exclusion (Williams, 2007) and a subsequent depletion of emotional resources (Shen et al., 2019). To cope with this perceived relational exclusion, marginalized members tend to engage in defensive behavioral withdrawal. Research suggests that social exclusion tends to reduce individuals' prosocial motivation and their willingness to offer reciprocal help to the collective (Twenge et al., 2007; Balliet and Ferris, 2013). Concurrently, “insiders” may hoard resources to protect their privileged status (Evans et al., 2015). This mutual withdrawal tends to erode the interpersonal trust and psychological safety required for effective team functioning (Edmondson, 1999). Because team creativity inherently involves risk-taking and learning from failure, it relies heavily on a supportive affective environment where members feel safe to experiment without fear of interpersonal penalty (Carmeli et al., 2010; Hu and Liden, 2015). By diminishing this essential affective support, TDA is expected to hinder team creativity. In sum, the following hypothesis is proposed:

H2: Team mutual collaboration mediates the relationship between team differential atmosphere and team creativity.

### 2.5 The mediating role of Team Joint Decision-Making

While Team Mutual Collaboration captures the team's affective bonds and interpersonal reciprocity, Team Joint Decision-Making represents a distinct cognitive and informational process. It entails the deliberate sharing, elaboration, and synthesis of diverse perspectives to reach consensus (Hinsz et al., 1997; van Knippenberg, 2017). TDA can impair team creativity by constraining this critical cognitive mechanism. From a SIPT perspective, the relation-based boundaries of TDA signal an environment characterized by high interpersonal risk (Edmondson, 1999). Decoding this cue, “outsiders” are more likely to engage in defensive cognitive withholding—such as employee silence—fearing that their unique viewpoints might be undervalued or attract interpersonal sanctions from the in-group (Morrison, 2014; Chen and Liu, 2020). Conversely, to maintain their status, “insiders” may dominate the discourse, which can further marginalize divergent cognitive inputs (Tost et al., 2013). Because team creativity, particularly in multicultural teams, depends on the cross-pollination and elaboration of diverse cultural and professional knowledge (De Dreu and West, 2001; Stahl et al., 2010), this defensive restriction of information flow can pose a notable barrier. Meta-analytic evidence suggests that limiting information sharing and cognitive integration tends to lower the quality of joint decision-making (Mesmer-Magnus and DeChurch, 2009), thereby limiting the cognitive resources available for creative output. In sum, the following hypothesis is proposed:

H3: Team joint decision-making mediates the relationship between team differential atmosphere and team creativity.

### 2.6 The moderating role of team empathy

This study posits that team empathy mitigates the negative effects of TDA on team interaction processes, acting as an important buffer in multicultural teams. Drawing on SIPT, we argue that team empathy operates as a team-level emotional–cognitive resource that shapes how members attend to and interpret the social cues associated with TDA (Salancik and Pfeffer, 1978; Akgün et al., 2015).

From an affective perspective, team empathy can weaken the impact of TDA on Team Mutual Collaboration. When team empathy is low, TDA-related cues (e.g., “Why did the leader favor that group?”) are more likely to be interpreted as signals of exclusion, especially along cultural faultlines, which increases feelings of unfairness and social distance and reduces members' willingness to help and cooperate with one another (Shin and Zhou, 2007). In contrast, when team empathy is high, members are more inclined to recognize and understand colleagues' emotional states, provide socio-emotional support, and maintain cooperative behaviors even when they perceive differential treatment (Rego et al., 2007; Bourke et al., 2020). By facilitating this more supportive interpretation and response, team empathy is expected to attenuate the negative relationship between TDA and team mutual collaboration. In sum, the following hypothesis is proposed:

H4a: Team empathy moderates the negative relationship between team differential atmosphere and team mutual collaboration, such that the relationship is weaker when team empathy is high.

Combining H2 and H4a, we further expect a moderated mediation effect. When team empathy is high, the extent to which TDA reduces mutual collaboration—and, in turn, team creativity—should be weakened (Bourke et al., 2020). In sum, the following hypothesis is proposed:

H4b: Team empathy moderates the indirect negative effect of team differential atmosphere on team creativity via team mutual collaboration, such that the negative indirect effect is weaker when team empathy is high.

Similarly, this study argues that Team Empathy mitigates the negative effect of TDA on Team Joint Decision-Making by influencing how diverse information is processed in multicultural teams. In low-empathy teams, TDA makes members focus more on who is favored, increasing the likelihood that the contributions of “out-group” members (often from minority cultural backgrounds) are discounted or ignored, and that divergent views are interpreted as challenges to status, which undermines information sharing and consensus building (Zaki, 2014). When Team Empathy is high, members are more likely to acknowledge the informational value of different perspectives, engage in perspective taking, and interpret disagreement as task-related input that can improve collective decisions rather than as a personal or status threat (Rego et al., 2007; Lee and Madera, 2021; Lee and Lim, 2023). This reduces the emotional volatility caused by TDA and allows the team to integrate diverse viewpoints into joint solutions without excessive interpersonal hostility (De Dreu et al., 2000; Fleischhauer et al., 2024). Thus, the negative relationship between TDA and Team Joint Decision-Making is expected to be weaker when Team Empathy is high. In sum, the following hypothesis is proposed:

H5a: Team empathy moderates the negative relationship between team differential atmosphere and team joint decision-making, such that the relationship is weaker when team empathy is high.

Combining H3 and H5a, we additionally expect that Team Empathy moderates the indirect effect of TDA on Team Creativity through Team Joint Decision-Making. When Team Empathy is high, the degree to which TDA undermines joint decision-making—and thereby team creativity—should be reduced (Zaki, 2014; Lee and Madera, 2021). In sum, the following hypothesis is proposed:

H5b: Team empathy moderates the indirect negative effect of team differential atmosphere on team creativity via team joint decision-making, such that the negative indirect effect is weaker when team empathy is high.

## 3 Research methods

### 3.1 Sample selection and data collection

This study employed a survey-based approach to collect data for testing the proposed research hypotheses. The sample primarily consisted of knowledge teams from 19 enterprises located in Shaanxi and Shandong provinces, including three main types of teams: research and development (R&D), design, and marketing management teams. First, Shaanxi and Shandong were selected as the two focal provinces for this study because their economic backgrounds, industry development levels, and cultural characteristics are broadly representative of many regions in China and are commonly used as research settings in Chinese management studies. These provinces encompass a wide range of industries, thereby enhancing the external validity of the research findings. Second, industries such as R&D, design, and marketing management face high levels of complexity and innovation demands. Teams in these industries typically engage in rich internal interactions, making them suitable for examining the relationships between the studied variables in a comprehensive manner.

The sample was selected using a stratified sampling approach based on enterprise and team characteristics. Specifically, we first identified strata according to enterprise size (small, medium, large), industry type (e.g., manufacturing, services, technology), and team function (R&D, design, marketing management). Within each stratum, teams that met the inclusion criteria were then randomly selected from the 19 enterprises to ensure the diversity of the sample. This procedure helped maintain balance across different team sizes and types and improved the overall representativeness of the sample.

To minimize common method bias, data were collected using a leader-member pairing approach, with some items designed as reverse-scored questions. Before distributing the questionnaires, the researchers explained the purpose of the study, the voluntary nature of participation, and confidentiality protections to all participants. Written informed consent was obtained from both team members and leaders. This study adopted a time-lagged data collection approach to further reduce the impact of common method bias. Data were collected at two time points. The first round of data collection was conducted from November 25 to 28, 2024, during which team members completed Questionnaire 1, including demographic information (e.g., gender, age, and education level) and measurements of team differential atmosphere and team empathy. The second round was conducted from December 28 to 30, 2024. During this stage, team members continued with Questionnaire 1, providing assessments of team mutual collaboration and joint decision-making, while team leaders completed Questionnaire 2, which included their demographic information as well as items regarding team size, team tenure, and team creativity. This data collection method ensured that team members' and leaders' perspectives were differentiated in the dataset, reducing self-reporting bias by leaders and improving the reliability and validity of the data.

A total of 98 teams were invited to participate in the study, including 98 team leaders and 536 team members. Completed responses were received from 85 teams, with 85 leaders and 385 team members participating in the survey. After screening and excluding invalid questionnaires with evident issues, a final sample of 79 teams was obtained, including 79 leader questionnaires and 356 member questionnaires, with an average of 4.51 team members per leader.

Leader sample: 62.03% male and 37.97% female; the average age was 36.29 years; 70.89% held a bachelor's degree or higher.

Employee sample: 47.75% male and 52.25% female; the average age was 30.56 years; 64.33% held a bachelor's degree or higher.

### 3.2 Variable measurement

All scales used in this study were adapted from well-established instruments (for the complete items, please refer to Supplementary File 1). After conducting focus group discussions with R&D teams and pilot tests with small samples, unreasonable items were removed, and wording was revised to form the final scales. All items were scored on a 7-point Likert scale, where 1 indicated “strongly disagree” and 7 indicated “strongly agree.”

(1) Team Differential Atmosphere. This variable was rated by subordinate team members and measured using 11 items based on the approach of Liu et al. (2009). The scale included three dimensions: “favoritism,” “interdependence,” and “insider roles.” An example item is: “The supervisor communicates information through specific subordinates.” In this study, the Cronbach's α for this scale was 0.964.

(2) Team Empathy. This variable was rated by subordinate team members and measured using 14 items adapted from Davis (1980), encompassing two dimensions: “cognitive empathy” and “emotional empathy.” An example item is: “When I see a team member being exploited, I feel a protective inclination toward them.” In this study, the Cronbach's α for this scale was 0.786.

(3) Team Mutual Collaboration. This variable was rated by subordinate team members and measured using three items adapted from Yao and Sun (2009). An example item is: “Team members voluntarily help busy colleagues complete their work.” Since team mutual collaboration is a team-level variable, individual-level data were aggregated to the team level to derive the score for mutual collaboration. The Cronbach's α for this scale in this study was 0.843.

(4) Team Joint Decision-Making. This variable was rated by subordinate team members and measured using three items adapted from Yao and Sun (2009). An example item is: “Team members collectively generate high-quality ideas.” As a team-level variable, individual responses were aggregated to the team level to calculate the score for joint decision-making. The Cronbach's α for this scale in this study was 0.879.

(5) Team Creativity. To mitigate potential common method bias, this variable was evaluated by the team supervisors and measured using three items adapted from Ng and Lucianetti (2016). An example item is: “Our team often thinks of new ideas to improve and enhance the current situation.” The Cronbach's α for this scale in this study was 0.740.

(6) Control Variables. Based on the work of Shen et al. (2019), commonly used demographic variables in organizational behavior research were selected as control variables: team members' gender, age, tenure, and educational level. Age was categorized into five groups: under 25, 26–35, 36–45, 46–55, and over 55. Tenure was divided into five ranges: less than 1 year, 1–3 years, 4–7 years, 8–10 years, and over 10 years. Educational level was classified into four stages: high school or below, associate degree, bachelor's degree, and master's degree or above.

## 4 Empirical analysis

### 4.1 Aggregation analysis

To ensure the methodological rigor of aggregating individual-level subordinate responses to the team level, this study strictly followed the typology of composition models proposed by Chan (1998) and Klein and Kozlowski (2000). For Team Differential Atmosphere (TDA), Team Mutual Collaboration, and Team Joint Decision-Making, we adopted the Referent-Shift Consensus Model (Chan, 1998). In this model, the referent of the survey items shifts from the individual to the collective (e.g., assessing how the supervisor treats the team, or how team members collaborate). Because these variables capture a shared team climate or collective process, it is methodologically required to demonstrate high within-team agreement. Therefore, before calculating the team-level mean scores for these three variables, we rigorously assessed their aggregation validity by calculating the within-group agreement (Rwg) and the intraclass correlation coefficients (ICC1 and ICC2). As shown in Table 1, the Rwg values for all three variables exceeded 0.70, indicating acceptable consistency. Furthermore, the results showed that the ICC (1) values for these variables exceeded the threshold of 0.12, and the ICC (2) values were above the critical value of 0.50, meeting the required standards. Consequently, the individual-level survey data for Team Differential Atmosphere, Team Mutual Collaboration, and Team Joint Decision-Making were validly aggregated to the team level for analysis. For Team Empathy, we applied the Additive Composition Model (Chan, 1998). Because the survey items for this construct focus on individual affective states, the team-level property is theoretically conceptualized as the sum or average of the lower-level units, regardless of the variance among them. Representing the collective pool of empathetic resources available within the team (Akgün et al., 2015), Team Empathy was appropriately operationalized by directly calculating the arithmetic mean of the team members' individual scores. Finally, regarding Team Creativity, this dependent variable was directly evaluated by the team supervisors. Because the supervisor provides a single, global assessment of the team's overall creative output, this construct is inherently captured at the team level and therefore does not require data aggregation procedures.

**Table 1:** Aggregate analysis results (*N* = 79).

**Variables**	**Rwg**	**ICC (1)**	**ICC (2)**
Team differential atmosphere	0.897	0.577	0.586
Team mutual collaboration	0.968	0.772	0.939
Team joint decision-making	0.978	0.701	0.795

### 4.2 Reliability, validity, and correlation analysis

First, the reliability analysis showed that the Cronbach's α values for all variables in this study exceeded 0.700, indicating high internal consistency across variables (see Table 2). Construct validity was used to evaluate the validity of the variable scales, comprising both convergent validity and discriminant validity. In terms of convergent validity, the composite reliability (CR) values for all variables exceeded the critical threshold of 0.70, and the average variance extracted (AVE) values were above the threshold of 0.50, demonstrating good convergent validity for all variables in this study (see Table 2).

**Table 2:** Validity of variables (*N* = 79).

**Variables**	**CR**	**AVE**
Team differential atmosphere	0.979	0.810
Team mutual collaboration	0.844	0.645
Team joint decision-making	0.885	0.533
Team empathy	0.952	0.870
Team creativity	0.757	0.513

Regarding discriminant validity, the square roots of the AVE values for each latent variable were greater than the correlation coefficients between the variable and other variables. Furthermore, to address potential conceptual overlap between the two mediators—Team Mutual Collaboration (TMC) and Team Joint Decision-Making (TJD)—we compared the hypothesized five-factor model with a four-factor model (merging TMC and TJD). Results from the Satorra-Bentler scaled chi-square difference test (TRd test) showed that the five-factor model fit the data significantly better [Δχ^2^ (6) = 378.56, *p* < 0.001], providing robust evidence for the distinctness of the two mediators.

Next, SPSS software was used to calculate the mean, standard deviation, reliability, and correlation coefficients of each variable. The specific results are shown in Table 3. As indicated in Table 3, the means and standard deviations of all variables fell within normal ranges, with no outliers observed. The results revealed significant negative correlations between team differential atmosphere and team creativity (*r* = –0.136, *p* < 0.05), team mutual collaboration (*r* = −0.525, *p* < 0.01), and team joint decision-making (*r* = −0.333, *p* < 0.01). Conversely, team mutual collaboration (*r* = 0.568, *p* < 0.01) and team joint decision-making (*r* = 0.521, *p* < 0.01) were significantly positively correlated with team creativity. These findings are consistent with the hypothesized directions and provide preliminary evidence supporting the research hypotheses.

**Table 3:** Mean, standard deviation, and correlation coefficient (*N* = 79).

**Var**	**Mean**	**SD**	**1**	**2**	**3**	**4**	**5**	**6**	**7**	**8**	**9**
Gen	0.504	0.350									
Age	2.518	0.566	−0.114								
Edu	2.629	0.702	−0.046	0.137							
Ten	2.686	0.869	0.323^**^	0.255^*^	−0.068						
TDA	4.617	1.019	0.07	−0.186	−0.027	−0.018	(0.900)				
TMC	5.272	0.798	−0.063	−0.027	0.017	−0.166	−0.525^**^	(0.803)			
TJD	5.202	0.607	−0.369^**^	0.243^*^	−0.122	−0.449^**^	−0.333^**^	0.395^**^	(0.730)		
TE	5.251	0.534	0.152	−0.159	−0.118	0.137	0.018	0.110	0.187	(0.933)	
TC	5.696	0.644	−0.118	0.150	−0.048	−0.284^*^	−0.136^*^	0.568^**^	0.521^**^	0.025	(0.716)

One asterisk (^*^): *p* < 0.05, indicating a significance level of 5%; Two asterisks (^**^): *p* < 0.01, indicating a significance level of 1%.

Var means Variables, Gen means Gender, Edu means Education Level, Ten means Tenure, TDA means Team Differential Atmosphere, TMC means Team Mutual Collaboration, TJD means Team Joint Decision-making, TE means Team Empathy, TC means Team Creativity.

### 4.3 Hypothesis testing

We used Mplus 7 to conduct a path analysis of the proposed moderated dual-mediation model. The results are shown in Table 4.

**Table 4:** Results of hierarchical regression analysis.

**Var**	**Team mutual collaboration**			**Team joint decision-making**			**Team creativity**		
	**M1**	**M2**	**M3**	**M4**	**M5**	**M6**	**M7**	**M8**	**M9**
Gen	−0.018	0.013	−0.048	−0.296	−0.321^*^	−0.312^*^	−0.046	−0.014	0.127
Age	0.020	−0.087	−0.039	0.407^***^	0.417^**^	0.437^***^	0.295^*^	0.294^**^	0.216
Edu	0.004	0.017	0.019	−0.187^*^	−0.164^*^	−0.171^*^	−0.098	−0.121	−0.039
Ten	−0.153	−0.164	−0.138^*^	−0.353^***^	−0.386^***^	−0.386^***^	−0.271^**^	−0.356^**^	−0.085
TDA		−0.424^***^	−0.499^***^		−0.159^***^	−0.208^***^		−0.218^*^	0.195^**^
TE		0.202	0.243		0.381^***^	0.408^***^			
TDA × TE			0.266^*^			0.173^*^			
TMC									0.484^***^
TJD									0.328^*^
R^2^	0.409^***^	0.587^***^	0.617^***^	0.028	0.331^***^	0.372^***^	0.145^*^	0.231^**^	0.497^***^
ΔR^2^	0.409^***^	0.178^***^	0.03^*^	0.028	0.303^**^	0.041^*^	0.145^*^	0.086^*^	0.266^***^

One asterisk (^*^): *p* < 0.05, indicating a significance level of 5%; Two asterisks (^**^): *p* < 0.01, indicating a significance level of 1%; Three asterisks (^***^): *p* < 0.001, indicating a significance level of 0.001.

Var means Variables, Gen means Gender, Edu means Education Level, Ten means Tenure, TDA means Team Differential Atmosphere, TMC means Team Mutual Collaboration, TJD means Team Joint Decision-making, TE means Team Empathy, TC means Team Creativity.

The analysis strongly supported the mediation hypotheses. First, team differential atmosphere had a significant negative direct effect on team creativity (β = –0.218, *p* < 0.05), supporting H1. For the mutual collaboration pathway, TDA significantly negatively predicted mutual collaboration (β = −0.424, *p* < 0.001), and mutual collaboration significantly positively predicted team creativity (β = 0.484, *p* < 0.001). A Bootstrap test revealed a significant indirect effect [Effect = −0.242, 95% CI = (−0.352, −0.152)], supporting H2.

For the joint decision-making pathway, TDA also significantly negatively predicted joint decision-making (β = –0.159, *p* < 0.001), and joint decision-making significantly positively predicted team creativity (β = 0.328, *p* < 0.05). The Bootstrap test showed that the indirect effect through joint decision-making was also significant [Effect = −0.068, 95% CI = (−0.161, −0.007)], supporting H3.

**Figure 2:**
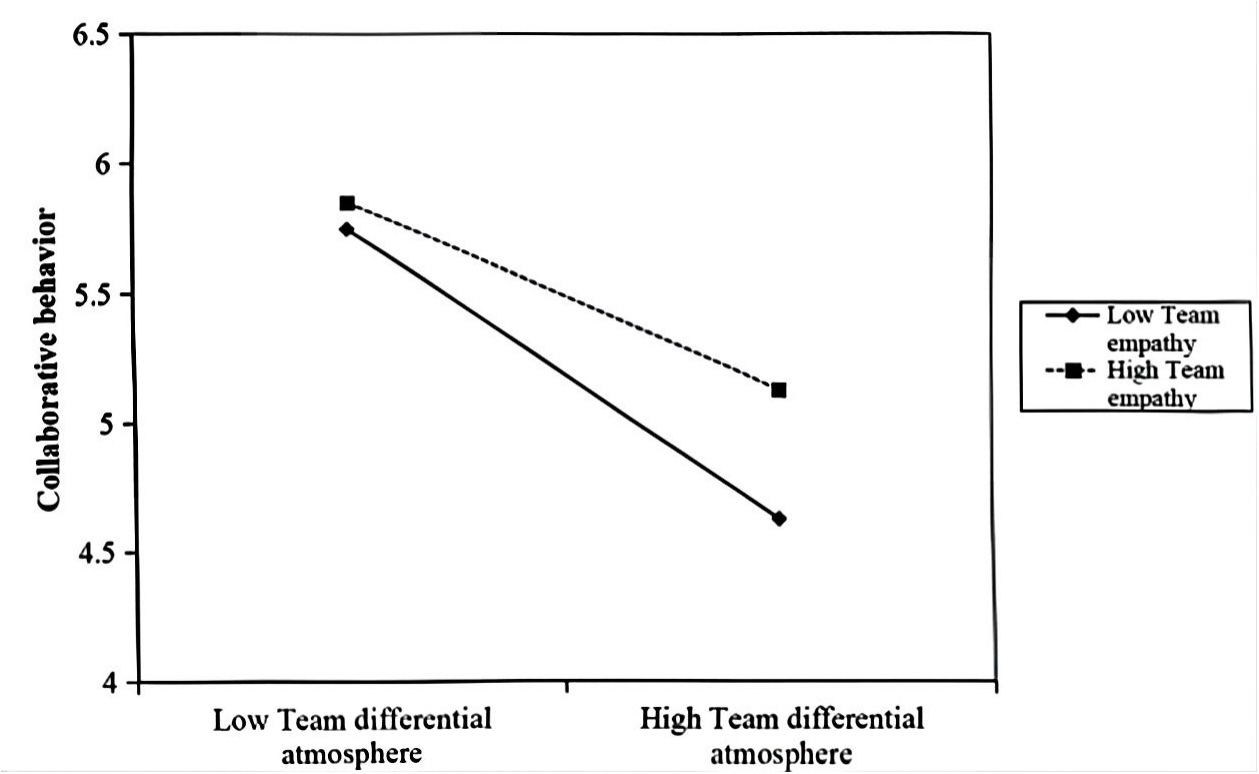
Moderating effect of team empathy on team mutual collaboration.

The results also supported the moderating role of team empathy. The interaction term of TDA and team empathy had a significant positive effect on mutual collaboration (β = 0.266, *p* < 0.05). A simple slope analysis showed that when team empathy was high, the negative effect of TDA on mutual collaboration behavior (simple slope = −0.357, *p* < 0.001) was weaker than when empathy was low (simple slope = −0.641, *p* < 0.001). This buffering effect is illustrated in Figure 2, supporting H4a.

**Figure 3:**
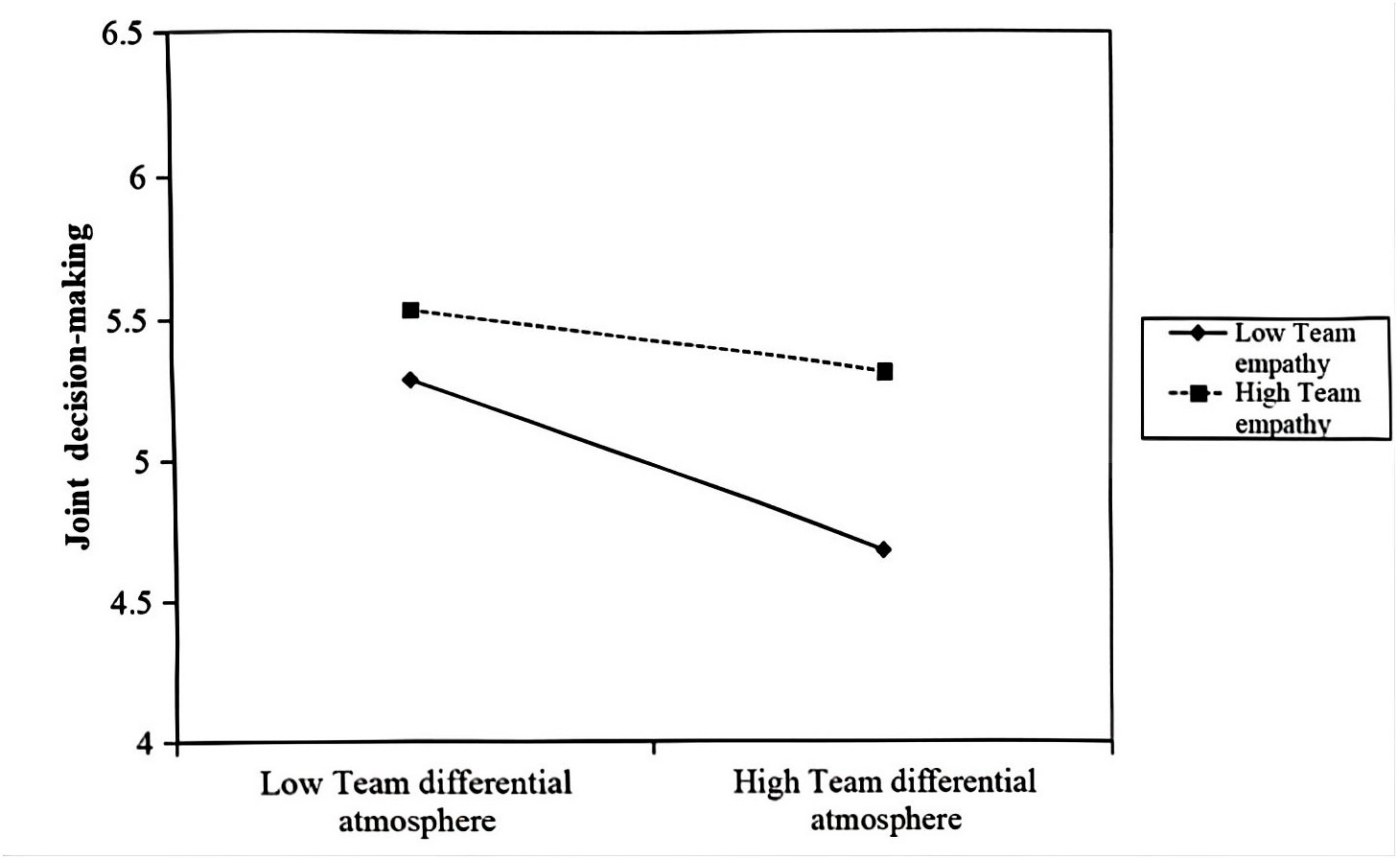
Moderating effect of team empathy on team joint decision-making.

Simultaneously, the interaction term of TDA and team empathy had a significant positive effect on joint decision-making (β = 0.173, *p* < 0.05). Simple slope analysis revealed that for teams with high empathy, the inhibitory effect of TDA on joint decision-making was weaker (simple slope = −0.116, p = 0.056), whereas for teams with low empathy, this negative effect was significantly stronger (simple slope = −0.300, *p* < 0.001). This buffering effect is depicted in Figure 3, supporting H5a.

Finally, we tested the overall moderated mediation model. As shown in Table 5, the results indicate that team empathy significantly moderated the strength of both mediation pathways.

**Table 5:** Results of moderated mediation analysis.

**Mediating variable**	**Level of moderator (team empathy)**	**Indirect effect (95% CI)**	**Index of mod. med. (95% CI)**
TMC	Low (−1SD)	−0.310 (−0.443, −0.184)	0.129 (0.031, 0.252)
	High (+1SD)	−0.173 (−0.261, −0.078)	
TJD	Low (−1SD)	−0.098 (−0.223, −0.004)	0.057 (0.002, 0.158)
	High (+1SD)	−0.038 (−0.098, 0.012)	

For the pathway through mutual collaboration, the index of moderated mediation was significant and positive [Index = 0.129, 95% Boot CI = (0.031, 0.252)]. The conditional indirect effect of TDA on team creativity via mutual collaboration was weaker at high levels of team empathy [indirect effect = −0.173, 95% CI (−0.261, −0.078)] and stronger at low levels [indirect effect = −0.310, 95% CI (−0.443, −0.184)]. Thus, H4b was supported.

For the pathway through joint decision-making, the index of moderated mediation was also significant and positive [Index = 0.057, 95% Boot CI = (0.002, 0.158)]. The conditional indirect effect of TDA on team creativity via joint decision-making was significantly weaker at high levels of empathy [indirect effect = −0.038, 95% CI (−0.098, 0.012)] than at low levels [indirect effect = −0.098, 95% CI (−0.223, −0.004)]. Thus, H5b was supported.

**Figure 4:**
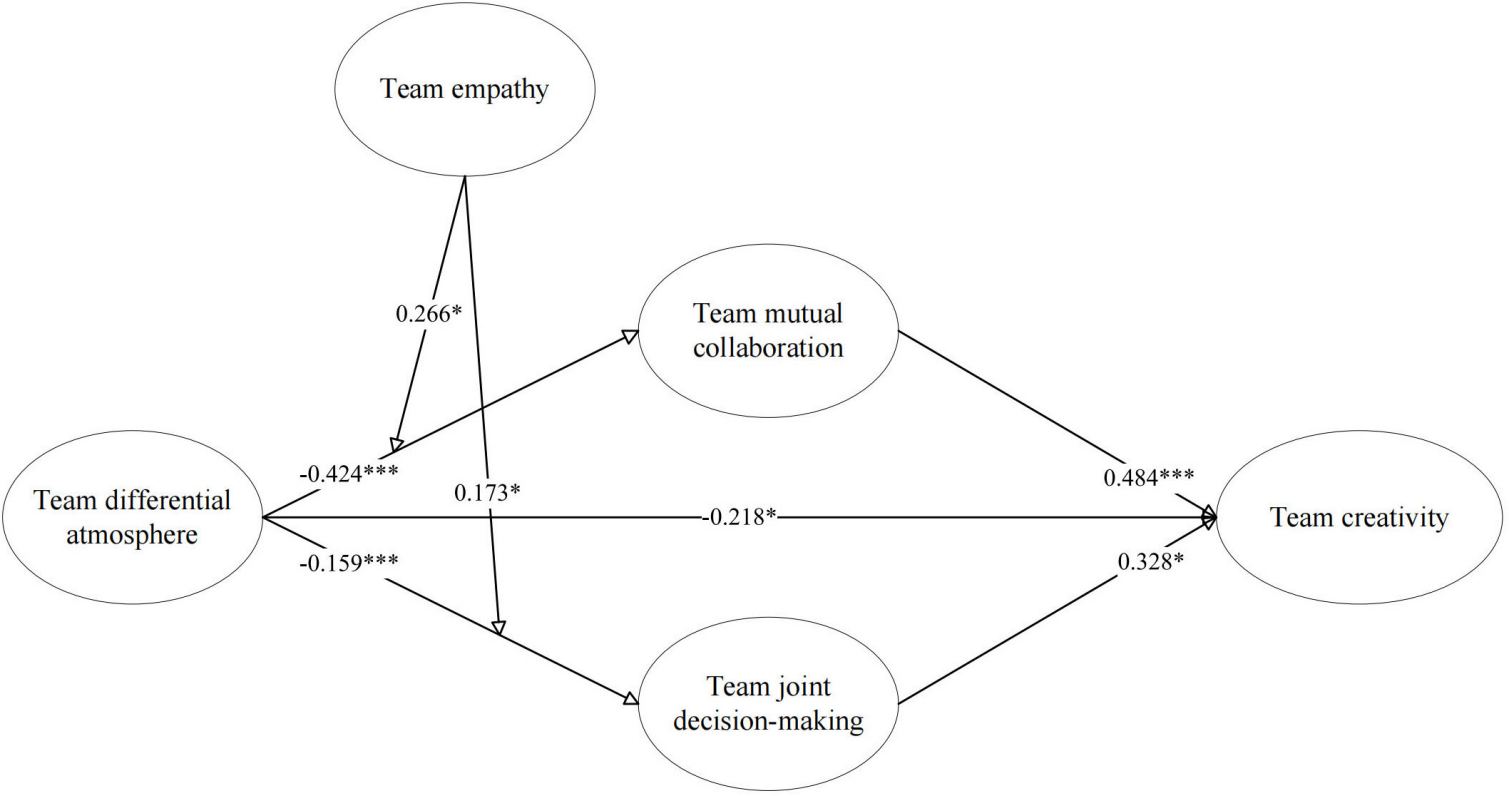
Path analysis results.

Figure 4 visually summarizes the coefficient test results of all hypothesized paths in the model.

## 5 Conclusion and discussion

Grounded in social information processing theory, this study aimed to investigate how and when a Team Differential Atmosphere—as a typical non-inclusive leadership behavior—inhibits team creativity, particularly within the increasingly prevalent context of multicultural teams. The findings confirm that a team differential atmosphere has a significant negative impact on team creativity. More importantly, we uncovered its underlying mechanism: this negative influence is realized by undermining two key team interaction processes—Team Mutual Collaboration and Team Joint Decision-Making. Furthermore, this study found that team empathy plays a crucial buffering role in this process: in high-empathy teams, the corrosive effects of a differential atmosphere on team collaboration and decision-making processes were significantly weakened. These findings collectively form a compelling narrative that an exclusionary atmosphere created by a leader systematically dismantles the collaborative foundation of a team, while the team's own emotional capability (empathy) serves as a key internal resource to counteract such negative leadership and protect innovative vitality.

It is important to acknowledge a nuanced finding from our interaction analysis (Figures 2, 3): while team empathy significantly flattens the negative slope of TDA, it does not render the relationship non-significant. In other words, even in high-empathy teams, high levels of TDA still exert a detrimental, albeit weaker, influence on team processes. This finding aligns with the structural primacy tenet of SIPT, which suggests that while social processing (e.g., empathy) can alter the interpretation of cues, it cannot entirely override the reality of strong structural signals. TDA, as a top-down allocation structure, creates objective resource disparities that ‘bottom-up' affective resources like empathy can buffer but not fully neutralize. This highlights that TDA is a potent toxic element in team dynamics that requires root-cause intervention from leadership, as relying solely on team resilience is insufficient.

### 5.1 Theoretical contribution

Within a refined Social Information Processing Theory (SIPT) framework (Salancik and Pfeffer, 1978), this study makes several key contributions to the literature on inclusive leadership, multicultural team management, and team creativity.

First, this study advances the theoretical understanding of non-inclusive leadership by strictly distinguishing the “identity signal” of Team Differential Atmosphere (TDA) from the “performance signal” of LMX Differentiation (LMXD). While previous research often conflated differential treatment with general unfairness, we apply a SIPT lens to clarify their distinct signaling mechanisms. We argue that unlike LMXD, which functions as a transactional “performance cue” rooted in social exchange (Liden et al., 2006), TDA emits a salient “identity threat cue” based on relational closeness and rigid “insider-outsider” boundaries (Liu et al., 2009; Shen et al., 2019). This distinction offers a novel theoretical perspective: TDA inhibits creativity not merely by creating resource disparities, but by triggering identity-defensive mechanisms that are qualitatively different from the competitive dynamics associated with LMXD. By placing TDA in dialogue with mainstream LMX theory, this study provides robust empirical evidence on why relational differentiation is particularly destructive in multicultural contexts.

Second, this study opens the “black box” of how TDA stifles team creativity by identifying two distinct mediating pathways: the erosion of behavioral bonds and the blockage of cognitive integration. Previous studies have largely treated team processes as a monolithic construct. Our research confirms that TDA exerts its negative influence by simultaneously undermining Team Mutual Collaboration (a behavioral mechanism involving reciprocal support and trust) and Team Joint Decision-Making (a cognitive mechanism involving the synthesis of diverse information) (Yao and Sun, 2009). The deeper implication is that TDA destroys the dual cornerstones of multicultural team success: it dissolves the “will” to cooperate and disables the “way” to integrate differences (Van Knippenberg and Schippers, 2007). This finding deepens the indigenous interpretation of SIPT, suggesting that in multicultural contexts, negative social cues are amplified to disrupt both the relational fabric and the cognitive processing capabilities of the team (Triana et al., 2021).

Third, this study establishes Team Empathy as a critical “bottom-up” emotional–cognitive resource that buffers the adverse effects of differential leadership. Moving beyond the “top-down” focus of traditional leadership research, we reveal the agency of the team itself. We confirm that Team Empathy acts as a vital resilience mechanism (Roberge, 2013; Akgün et al., 2015). In high-empathy teams, members utilize perspective-taking to reframe the exclusion signals emitted by TDA, interpreting them less as identity threats and more as structural challenges, thereby maintaining necessary cooperation and information sharing (Rego et al., 2007; Zaki, 2014). Crucially, rather than relying solely on leadership interventions, we highlight that the team's collective capacity for empathy serves as an internal buffer. This discovery provides a novel affective perspective on how teams can internally maintain psychological safety and withstand the friction caused by divisive leadership styles.

### 5.2 Practical implications

The findings of this study offer the following actionable managerial implications for organizations, particularly for leaders managing increasingly diverse teams:

First, leaders must be vigilant against and proactively manage “unconscious bias” to cultivate a fair and inclusive team atmosphere. The formation of a differential atmosphere often stems from a leader's unconscious affinity bias, which creates rigid relational boundaries (Bourke and Dillon, 2016). Therefore, organizations should provide managers with training on identifying and managing unconscious bias to help them reflect on their own biased behaviors (Dobbin and Kalev, 2017). More importantly, it is essential to establish and strictly adhere to transparent, performance-based decision-making mechanisms. By replacing relational cues with structural fairness, organizations can curb the “identity threat” signal of TDA and build an inclusive foundation for all members (Bohnet, 2016).

Second, managers should make building “psychological safety” and promoting “inclusive team interaction processes” a core responsibility. Our research shows that a differential atmosphere inhibits creativity by simultaneously undermining behavioral collaboration and cognitive decision-making. Therefore, a leader's focus should shift from simple task management to meticulous process design. Managers need to use institutional design and cultural guidance to ensure communication channels are open to everyone, and to encourage and protect the expression of dissenting opinions, making every member feel their voice is valued (Edmondson, 1999; Randel et al., 2018). This is crucial for unlocking the innovative potential of multicultural teams.

Third, organizations should place a high priority on systematically cultivating team empathy, treating it as a fundamental emotional–cognitive resource. Leaders themselves should lead by example, demonstrating empathy in their daily work (Goleman, 2016). Concurrently, research indicates that empathy is a skill that can be enhanced through training and practice (Riess, 2017). Organizations can raise team empathy levels through team-building exercises and cross-cultural communication workshops. This not only buffers the impact of negative leadership behaviors but also fundamentally strengthens the team's internal resilience and cohesion, allowing members to navigate differences more effectively.

### 5.3 Limitations and future directions

Although this study has made certain advancements, it still has the following limitations that warrant further investigation in future research.

First, the sample for this study was sourced exclusively from innovation teams in a specific region of China, which may limit the generalizability of the findings. As a construct with deep cultural imprints, the mechanism through which a differential atmosphere affects team creativity might vary across different cultural contexts. Therefore, future research could conduct cross-cultural comparisons to test the applicability and uniqueness of this study's model in Western or other cultural settings. For instance, would the detrimental effect of TDA on team interaction processes be stronger or weaker in individualistic cultures compared to collectivistic ones.

Second, although the two-wave data collection design used in this study controlled for common method bias to some extent, it still cannot fully establish causal relationships between variables, nor can it rule out the potential influence of external shocks (such as organizational strategy adjustments) (van Knippenberg, 2017). Future studies could employ multi-wave longitudinal designs or use experimental methods to manipulate key variables, thereby more rigorously revealing the dynamic process and causal chain of how a team differential atmosphere affects team creativity.

Third, the measurement of team creativity relied on the single-source evaluation of the team leader. Although this method is common in the field (Ng and Lucianetti, 2016; Chen and Liu, 2020), it may still be influenced by subjective factors such as the leader's personal preferences or social desirability bias. Future research could incorporate multi-source evaluation systems, such as peer ratings from team members, assessments by external experts, or even more objective indicators of innovative output, to provide a more comprehensive evaluation of team creativity.

Finally, the model in this study could be further expanded. Future research could explore additional boundary conditions affecting this process. For instance, could a leader's own pro-diversity beliefs (Randel et al., 2018) or cultural intelligence (Jonsen et al., 2021) effectively prevent the formation of a differential atmosphere. Could clear organizational DEI (Diversity, Equity, and Inclusion) policies and culture buffer the negative impact of TDA on the team. Investigating these questions will help build a more complete theoretical framework on how to lead effectively in a multicultural world.

## Data Availability

The raw data supporting the conclusions of this article will be made available by the authors, without undue reservation.
