# Supplementary material for: The dark side of team dynamics: how team differential atmosphere undermines team creativity
Source: Front Psychol. 2026 Mar 25;17:1658508. doi: 10.3389/fpsyg.2026.1658508 (PMC13056854; doi:10.3389/fpsyg.2026.1658508)
Supplement: Supplementary file 1 [file Supplementary_file_1.docx]

Supplementary Material

# Variable Measurement Item

## Team Differential Atmosphere

1. Supervisors have frequent contact with individual subordinates.

2. The supervisor shares his ideas and practices with a fixed number of subordinates on the team.

3. In the team, I believe that certain subordinates have a strong influence on the manager's decisions.

4. In the team, the supervisor has a close relationship with individual subordinates

5. Supervisors use specific subordinates to get their messages across.

6. On the team, the supervisor has subordinates whom he trusts in particular.

7. In the team, I feel that the supervisor treats his subordinates more differently.

8. Some subordinates were promoted much faster than others.

9. Supervisors often leave personal matters to individual subordinates.

10. There are certain subordinates who assist the supervisor with routine tasks.

11. There are certain subordinates who fill in most of the supervisor's duties.

## Team Collaborative Behavior

1. Team members volunteer to help busy members get work done.

2. Team members adjust tasks flexibly to make it easier to work with each other.

3. Team members help each other to complete work on time.

## Joint Decision-making

1. Team members produce quality ideas together.

2. Team members can come up with quality solutions together.

3. Team members are able to make high-level creative and innovative decisions together.

## Team Empathy

1. When I see team members being used, I develop protective tendencies toward them.

2. When I see team members being treated unfairly, I sometimes don't feel sorry for them. R

3. I often have warm, caring feelings toward those less fortunate than me on my team.

4. I would describe myself as a soft-hearted person.

5. I sometimes don't feel sorry for my team members when they have problems.

6. Team members' misfortunes usually don't confuse me. R

7. I am often moved by the things I see.

8. Before criticizing team members, I try to imagine how I would feel if I were in their place.

9. If I'm sure I'm right about something, I don't waste too much time listening to other people's opinions.

10. I sometimes try to get to know team members better by imagining things from their point of view.

11. I believe there are two sides to every story and try to see them at the same time.

12. I find it sometimes difficult to see things from someone else's point of view. R

13. I try to understand everyone's differences before making a decision.

14. When I'm disappointed in someone, I usually try to put myself in that person's shoes for a while.

## Team Creativity

Appendix A.5 Team Creativity.

1. Our team comes up with new ideas to improve and improve the status quo.

2. Our team looks for new working methods, techniques and tools.

3. Our team will find new ways to solve problems.
